# Surgical and short-term outcomes in robotic and laparoscopic distal gastrectomy for gastric cancer with enhanced recovery after surgery protocol: A propensity score matching analysis

**DOI:** 10.3389/fsurg.2022.944395

**Published:** 2022-10-06

**Authors:** Weijia Huang, Siyu Liu, Junqiang Chen

**Affiliations:** ^1^ Department of Gastrointestinal Gland Surgery, The First Affiliated Hospital of Guangxi Medical University, Nanning, China; ^2^ Guangxi Laboratory of Enhanced Recovery after Surgery for Gastrointestinal Cancer, The First Affiliated Hospital of Guangxi Medical University, Nanning, China; ^3^ Guangxi Clinical Research Center for Enhanced Recovery after Surgery, The First Affiliated Hospital of Guangxi Medical University, Nanning, China; ^4^ Guangxi Zhuang Autonomous Region Engineering Research Center for Artificial Intelligence Analysis of Multimodal Tumor Images, The First Affiliated Hospital of Guangxi Medical University, Nanning, China

**Keywords:** enhanced recovery after surgery, gastric cancer, robot distal gastrectomy, laparoscopy distal gastrectomy, propensity score matching

## Abstract

**Objective:**

This study aims to evaluate the short-term surgical outcomes of laparoscopy-assisted distal gastrectomy (LADG) and robot-assisted distal gastrectomy (RADG) for gastric cancer (GC) with enhanced recovery after surgery (ERAS) protocols.

**Methods:**

We reviewed the medical records of 202 patients undergoing radical distal gastrectomy; among them, 67 cases were assisted through RADG, while 135 cases were assisted through LADG along with ERAS. We retrospectively collected the medical records in succession from a database (January 2016–March 2019). We adopted propensity score matching to compare surgical and short-term outcomes of both groups.

**Results:**

After the successful examination of 134 cases, including 67 receiving RADG and 67 undergoing LADG, the operative times were noted as 5.78 ± 0.96 h for the RADG group and 4.47 ± 1.01 h for the LADG group (*P* < 0.001). The blood loss was noted as 125.52 ± 101.18 ml in the RADG group and 164.93 ± 109.32 ml in the LADG group (*P* < 0.05). The shorter time to first flatus was 38.82 ± 10.56 h in the RADG group and 42.88 ± 11.25 h in the LADG group (*P* < 0.05). In contrast, shorter days of postoperative hospital stay were 5.94 ± 1.89 days in the RADG group and 6.64 ± 1.92 days in the LADG group (*P* < 0.05). Also, the RADG group (84483.03 ± 9487.37) was much more costly than the LADG group (65258.13 ± 8928.33) (*P* < 0.001). The postoperative overall complication rates, numbers of dissected lymph nodes, visual analogue scale (VAS), and time to start a liquid diet for the RADG group and the LADG group were similar.

**Conclusions:**

In this research, we concluded that RADG provides surgical benefits and short-term outcomes compared to LADG for GC with ERAS.

## Introduction

Gastric cancer (GC) is a frequently occurring cancer of the digestive system globally, and it ranks third according to morbidity and mortality rates in China (1). In 1990, Kehlet first proposed the concept of enhanced recovery after surgery (ERAS) (2). ERAS is a comprehensive multidisciplinary treatment through a series of intervention measures to optimize perioperative treatment and reduce the physical and psychological trauma pressure of patients. It enhances the improvement of patients with prognostic outcomes and reduces the length of stay. After more than 10 years of development, ERAS is under much acknowledgment and has been adopted in multiple surgical specialties. The formulation of consensus guidelines for ERAS filled the gap of an ERAS protocol for treating GC (3). In addition, Kitano was the first to propose the concept of laparoscopy-assisted gastrectomy (LAG) in 1994; it can significantly shorten the operation time and reduce postoperative complications compared to open surgery (4, 5). With the innovation and progress of minimally invasive technology, the da Vinci robotic surgery system is extensively utilized clinically. The robot has higher magnification, stereoscopic vision, more flexible instruments, and filter tremors compared to laparoscopy (6, 7). To date, most studies on robot-assisted gastrectomy (RAG) and LAG to treat GC along with ERAS protocols are retrospective studies. Consequently, propensity score matching (PSM) was adopted for evaluating the short-term outcomes of LAG and RAG to treat GC with ERAS protocols (8).

## Materials and methods

### Study population

This research was approved by the Institutional Review of the First Affiliated Hospital of Guangxi Medical University. From 2016 to 2019, we performed a total of 67 robot-assisted distal gastrectomy (RADG) and 135 laparoscopy-assisted distal gastrectomy (LADG) surgeries in GC cases receiving our complete version of ERAS. The components of ERAS are summarized in Table 1. Selected patients should meet the following criteria: the patient decided to accept the ERAS program treatment after an informed discussion and have a high degree of compliance. All cases were diagnosed before surgery through CT and upper endoscopy. All patients underwent distal gastrectomy. Patients have no conversion to open gastrectomy, no other malignancy or distant metastasis, and no neoadjuvant chemotherapy or radiotherapy before the operation. Patients have complete clinical data.

**Table 1:** Components of an ERAS protocol.

ERAS category	Component
Preoperative	Preadmission education about ERAS
	No smoking, no alcohol, respiratory function training if necessary
	Carbohydrate drink until 10 and 2 h before surgery
	No bowel preparation
	No diazepam sedatives
	Antibiotic prophylaxis half an hour before the operation
Intraoperative	Optimize the anesthetic program (general anesthesia combined with epidural anesthesia)
	Minimally invasive incisions (preference for laparoscopic or robotic surgery)
	Active warming (aim for body temperature of 37 °C)
	Preventive analgesia (TAP, PCA, and subcutaneous injection of ropivacaine)
	Avoidance of nasogastric tubes and drains (if used, early removal)
	Liquid management (choosing vasoconstrictor drugs to control blood pressure)
Postoperative	Postoperative antiemetic (tropisetron hydrochloride, IV BID)
	Pain management (continue to place PCA, flurbiprofen axetil injection IV BID), VAS is performed daily
	Urinary catheter removed on first postoperative day, early nasogastric tube and drain removal
	Oral diet initiated from saline to liquid diet to semi-liquid diet, use pharmacological nutrients if necessary
	First postoperative day: 15–30 ml of normal saline was administered; thereafter, a liquid meal (rice soup, short peptides, total nutrients, glutamine, water-soluble vitamins, salt) of 15–30 ml was provided. In the second meal, after gastrointestinal tolerance, a liquid meal of 50–80 ml was dispensed
	Second postoperative day: a liquid meal of 80–100 ml was dispensed (rice soup, short peptides, total nutrients, glutamine, multivitamins, trace elements, salt); if no adverse reactions, adjustment continued gradually to 100–150 ml, q4h
	Third postoperative day: liquid diet + semi-liquid diet
	Fourth postoperative day: liquid diet + semi-liquid diet, gradually increasing the proportion of the semi-liquid diet
	Fifth postoperative day: gradually transition to a semi-liquid diet
	Monitoring of blood glucose, TID
	Promote the recovery of intestinal function (lactulose oral solution 15 ml, two or three times a day)
	Early ambulation (active mobilization in bed at least 6 h on first postoperative day, ambulation out of bed at least 2 h on second postoperative day)
	Defined discharge criteria
	Quality of life evaluation by the QoR-40 scale
Discharge criteria	For the recovery of bowel function, there is no need for intravenous fluids. Oral feeding can reach the preoperative intake level of 70%
	No analgesia was required, or the patient’s pain control was tolerable without intravenous analgesia or oral analgesics alone (VAS pain score ≤4)
	Able to complete daily activities and take care of themselves
	Pulse, blood pressure, heart rate, and body temperature should remain stable and at the same level as before surgery. Biochemical parameters, such as white blood cell count and hemoglobin, should be stable and maintained within a reasonable range
	Patients are willing to leave the hospital

ERAS, enhanced recovery after surgery; IV, intravenous; VAS, visual analogue scale; PCA, patient controlled analgesia; TAP, transversus abdominis plane block; QoR-40, quality of recovery-40 questionnaire.

### Outcomes

The patient outcomes and operative factors include time to start a liquid diet, time to first flatus, postoperative hospital stay, postoperative complication, numbers of dissected lymph nodes, visual analogue scale (VAS), estimated blood loss, operative time, and total cost. We applied the Clavien–Dindo classification to review the severity and frequency of occurrence of operative complications (9).

### Surgery

All surgeries were performed by the same surgical team. With a doctor’s degree and a senior professional title, the surgeons have completed more than 1,000 laparoscopic gastric cancer surgeries. In addition, the surgeons completed robotic surgery training. RADG and LADG were performed with a small laparotomy, and the reconstruction method was Billroth-II with Braun anastomosis. After comprehensive discussions, the patients chose the type of surgery according to possible risks and merits associated with RADG and LADG.

### Propensity score matching

We used PSM in SPSS (version 24.0) to match the two groups on a 1:1 basis and a 0.02 caliper width. The matching variables for PSM included demographics [age, gender, and body mass index (BMI)], American Society of Anesthesiologists (ASA) classification, infusion volume, tumor size, clinical stage, tumor site, and histologic differentiation.

### Statistical analysis

We used SPSS (version 24.0, IBM SPSS, Chicago, United States) for data analysis. We applied McNemar’s test, Pearson’s chi-square test, Mann–Whitney *U* test, and Fisher’s exact test for comparative analysis among different groups. A difference of *P* < 0.05 indicates statistical significance.

## Results

### Patient characteristics

The demographic profile of patients is shown in Table 2. Prior to PSM, differences in the clinical stage (*P* = 0.015) and BMI (*P* = 0.001) were of statistical significance between RADG and LADG groups. Following PSM, patient distributions between matched pairs were balanced. Both groups contained 67 cases for further investigations.

**Table 2:** Baseline characteristics of the RADG and LADG groups, before and after weighing.

Patient characteristics	All patients			Propensity-matched patients		
	RADG	LADG	*P*	RADG	LADG	*P*
	(*n* = 67)	(*n* = 135)		(*n* = 67)	(*n* = 67)	
Gender, No. (%)			0.289			0.581
Male	43 (64.2)	75 (55.6)		43 (64.2)	47 (70.1)	
Female	24 (35.8)	60 (44.4)		24 (35.8)	20 (29.9)	
Age, year	49.34 ± 12.42	50.63 ± 12.15	0.483	49.34 ± 12.42	49.15 ± 10.89	0.924
BMI (kg/m^2^)	22.50 ± 2.92	20.43 ± 2.17	**0.001**	22.50 ± 2.92	22.34 ± 2.49	0.875
ASA classification—no. (%)			0.315			0.735
I	4 (6.0)	10 (7.4)		4 (6.0)	3 (4.47)	
II	59 (88.0)	116 (85.9)		59 (88.0)	61 (91.06)	
III	4 (6.0)	9 (6.7)		4 (6.0)	3 (4.47)	
Infusion volume	2852.61 ± 729.158	2812.72 ± 787.358	0.729	2852.61 ± 729.158	2785.55 ± 692.81	0.586
Tumor size (cm)	3.78 ± 2.58	3.79 ± 2.39	0.980	3.78 ± 2.58	3.77 ± 2.85	0.985
Clinical stage, No. (%)			**0.015**			0.952
I	27 (40.3)	31 (23.0)		27 (40.3)	26 (38.8)	
II	19 (28.4)	36 (26.7)		19 (28.4)	21 (31.3)	
III	21 (31.3)	68 (50.4)		21 (31.3)	20 (29.9)	
Histology, No. (%)			0.199			0.746
Well differentiated	0 (0)	2 (1.5)		0 (0)	0 (0)	
Moderately differentiated	18 (26.9)	26 (19.2)		18 (20.9)	15 (22.4)	
Poorly differentiated	48 (71.6)	101 (74.8)		48 (79.1)	51 (76.1)	
Undifferentiated	1 (1.5)	6 (4.5)		1 (1.5)	1 (1.5)	
Tumor site, No. (%)			0.246			0.398
Lower	52 (77.6)	114 (84.4)		52 (77.6)	56 (83.6)	
Middle	15 (22.4)	21 (15.6)		15 (22.4)	11 (16.4)	

BMI, body mass index; ASA, American Society of Anesthesiologists; RADG, robot-assisted distal gastrectomy; LADG, laparoscopy-assisted distal gastrectomy.

Bold values are statistically significant (*P*** **< 0.05).

### Perioperative outcomes

Table 3 presents the details of the perioperative outcomes. RADG group had an increased average operative time compared to the LADG group (5.78 ± 0.96 vs. 4.47 ± 1.01 h, *P* < 0.001). In addition, the RADG group had reduced average approximated blood loss relative to the LADG group (125.52 ± 101.18 vs. 164.93 ± 109.32 ml, *P* = 0.032). However, cost analyses showed a higher total cost (84,483.03 ± 9,487.37 vs. 65,258.13 ± 8,928.33, *P* < 0.001). The RADG group had reduced postoperative hospital stay (5.94 ± 1.89 vs. 6.64 ± 1.92 days, *P* = 0.035) and time to the first flatus (38.82 ± 10.56 vs. 42.88 ± 11.25 h, *P* = 0.033) compared to the LADG group. The differences in time to the first liquid diet (*P* = 0.363) and numbers of dissected lymph nodes (*P* = 0.277) were not significant between the two groups.

**Table 3:** Perioperative and short-term oncologic outcomes between the RADG and LADG groups.

Patient characteristics	All patients			Propensity-matched patients		
	RADG	LADG	*P*	RADG	LADG	*P*
	(*n* = 67)	(*n* = 135)		(*n* = 67)	(*n* = 67)	
Lymph node harvest	29.39 ± 12.10	27.11 ± 13.11	0.235	29.39 ± 12.10	26.94 ± 13.80	0.277
Operative time (h)	5.78 ± 0.96	4.57 ± 1.05	**<0.001**	5.78 ± 0.96	4.47 ± 1.01	<**0.001**
Estimated blood loss (ml)	125.52 ± 101.11	163.37 ± 108.78	**0.028**	125.52 ± 101.11	164.93 ± 109.32	**0.032**
Time to first flatus (h)	38.82 ± 10.56	43.92 ± 11.94	**0.021**	38.82 ± 10.56	42.88 ± 11.25	**0.033**
Time to start a liquid diet (h)	37.28 ± 11.70	39.34 ± 11.61	0.334	37.28 ± 11.70	39.06 ± 10.81	0.363
Postoperative hospital stay (days)	5.94 ± 1.89	6.66 ± 1.96	**0.033**	5.94 ± 1.89	6.64 ± 1.92	**0.035**
Total cost (CNY)	84483.03 ± 9487.37	64553.04 ± 8295.69	<**0.001**	84483.03 ± 9487.37	65258.13 ± 8928.33	<**0.001**

RADG, robot-assisted distal gastrectomy; LADG, laparoscopy-assisted distal gastrectomy.

Bold values are statistically significant (*P* < 0.05).

Table 4 shows the results of the VAS of the patients. Both groups had similar VAS on the postoperative day, i.e., first, second, third, and fourth day (1.46 ± 0.82 vs. 1.57 ± 0.85 days, *P* = 0.473; 0.72 ± 0.77 vs. 0.72 ± 0.71 days, *P* = 1.000; 0.22 ± 0.55 vs. 0.21 ± 0.48 days, *P* = 0.866; 0.03 ± 0.17 vs. 0.04 ± 0.12 days, *P* = 0.758).

**Table 4:** Postoperative VAS between the RADG and LADG groups.

VAS	All patients			Propensity-matched patients		
	RADG	LADG	*P*	RADG	LADG	*P*
	(*n* = 67)	(*n* = 135)		(*n* = 67)	(*n* = 67)	
First postoperative day	1.46 ± 0.82	1.51 ± 0.82	0.716	1.46 ± 0.82	1.57 ± 0.85	0.473
Second postoperative day	0.72 ± 0.77	0.77 ± 0.74	0.644	0.72 ± 0.77	0.72 ± 0.71	1.000
Third postoperative day	0.22 ± 0.55	0.28 ± 0.56	0.534	0.22 ± 0.55	0.21 ± 0.48	0.866
Fourth postoperative day	0.03 ± 0.17	0.04 ± 0.22	0.712	0.03 ± 0.17	0.04 ± 0.12	0.758

VAS, visual analogue scale; RADG, robot-assisted distal gastrectomy; LADG, laparoscopy-assisted distal gastrectomy.

### Postoperative complications

The postoperative complications and the related degrees of the two groups are given in Table 5. There was no significant difference in the postoperative overall complication rate between both groups (4.5% vs. 5.9% *P* = 1.000).

**Table 5:** Postoperative complications.

Characteristics	All patients			Propensity-matched patients		
	RADG	LADG	*P*	RADG	LADG	*P*
	(*n* = 67)	(*n* = 135)		(*n* = 67)	(*n* = 67)	*** ***
Postoperative complication						
Chyle leak	1	0	1.000	1	0	1.000
Pulmonary infection	1	2	1.000	1	2	1.000
Gastroparesis	1	2	1.000	1	1	1.000
Anastomotic bleeding	0	1	1.000	0	1	1.000
Incision infection	0	1	1.000	0	0	1.000
Overall complications (%)	3 (4.5%)	6 (4.4%)	1.000	3 (4.5%)	4 (5.9%)	1.000
Clavien–Dindo classification						
Grade I (%)	0	1	1.000	0	0	—
Grade II (%)	2	3	1.000	2	3	1.000
Grade III (%)	1	1	1.000	1	1	1.000
Grade ≥ IV (%)	0	0	—	0	0	—

RADG, robot-assisted distal gastrectomy; LADG, laparoscopy-assisted distal gastrectomy.

## Discussion

A vital component of ERAS to treat GC is applying minimally invasive techniques, which have been greatly advocated by the ERAS Society (3). Radical gastrectomy with minimally invasive technologies, such as robotic or laparoscopic surgery, offers the advantages of small incisions, less bleeding, and less intraoperative injury; as a result, it reduces the surgical stress response.

In this study, the RADG group had markedly increased operative time compared with the LADG group. It might be due to the increase in the robot operating system. Another critical factor was the learning curve of RADG. In a study, the comparison of the first 100 patients with the subsequent 136 patients indicated that the average operative time decreased from 231 min at the start to 208 min later (10). As robotic technology develops and the gastrectomy experience accumulates, the time of robot-assisted gastrectomy will be significantly reduced. The RADG group had increased total hospitalization costs compared with the LADG group. In China, the National Medical Insurance System covers part of the cost of LADG but none of the RADG. Our result was consistent with several previous retrospective studies (11, 12).

Blood loss is considered an important parameter in assessing the safety and success rate of surgery. A study showed that perioperative blood transfusion might be a negative factor in oncologic outcomes (13). Our finding revealed that blood loss was decreased in the RADG group compared to that in the LADG group. Robotic surgery displays distinct over laparoscopic surgery, such as legible 3D images, a 10-fold magnified surgical field of vision, and seven degrees of freedom. With a larger field of view, clear 3D vision, and the ability to filter hand tremors, the robotic arm can reveal anatomical structures more accurately. This largely avoids vascular damage and provides stable hemostatic pressure for better hemostasis (14). In addition, the application of robotic systems can offer a better operating environment for surgeons to conduct minimally invasive operations (15).

More lymph nodes are needed for determining the GC stage and evaluating the prognosis more accurately. D2 lymph node dissection is suggested to improve the prognostic outcome of advanced GC (16). According to our results, both groups had comparable lymph nodes dissected. The current study demonstrated that LADG still has technical limitations included restricted freedom of movement of equipment. At the same time, RAG has a unique 3D operating system and a robotic arm that rotates freely at 360° as compared to LADG. RADG group was able to collect more lymph nodes during dissection of the D2 area (17, 18). Meanwhile, a previous report points out that in D2 lymph node dissection, robotic surgery has an advantage over lymph nodes in the common hepatic artery (no. 8), the para-celiac artery (no. 9), the proximal splenic artery (no. 11p), and the superior mesenteric vein (no. 14v), which can translate into potential survival benefits for patients (19).

The RADG group had earlier anal exhaust and shorter postoperative hospitalization time compared to the LADG group. The postoperative inflammatory response is an essential factor affecting the recovery of gastrointestinal function (20). In gastrectomy, the postoperative inflammatory response is mainly caused by the traction of organs and intraoperative injury. Robotic surgery can provide a larger and clearer surgical field of vision and more accurate and flexible operating angle and space, significantly reduce the operative damage caused by human factors with a less inflammatory response, and recover gastrointestinal function more quickly (21). GC recovers quickly following the radical operation, owing to the digestive tract reconstruction *via* endoscopic suture during the revolutionary process of GC. Thus, the incision is smaller that offers the merits of minimally invasive techniques, which dramatically alleviates pain and inflammation and reduces operative stress (22). The gastrointestinal function of the RADG group quickly recovered, along with a shorter time from a liquid diet to a semi-liquid diet and early discharge criteria (23).

Pain is one of the main reasons of stress caused after gastric cancer surgery (24); postoperative pain in patients undergoing radical gastrectomy is mainly caused by abdominal incision pain and visceral involvement pain. The use of multimodal analgesia is an essential part of ERAS. Adequate pain control can improve postoperative patients’ comfort and lead to earlier effective postoperative mobilization. In this study, perioperative pain intervention in ERAS included transversus abdominis plane, patient controlled analgesia, subcutaneous ropivacaine infiltration for prophylactic analgesia, and intravenous injection of acetaminophen or oral aminophenol hydrocodone for standardized analgesia from the date of surgery. From the first postoperative day to the fourth day, differences in VAS showed no significance between both groups.

Postoperative complications are important indexes of the short-term prognosis of GC. Our results suggested that the overall postoperative complications of RADG and LADG were 4.5% and 5.9%, respectively, with no statistically significant difference. At present, there is controversy in the research on the complication of RADG and LADG. Some researchers suggest that robots can perform more thorough lymph node dissection without damaging vital organs such as the spleen, pancreas, and large blood vessels. It is also believed that tumors can be removed and the digestive tract can be reconstructed with smaller incisions, reducing the risk of complications compared to laparoscopic surgery (25, 26). However, several studies have revealed that differences in postoperative complication rates are not significant between the two types of surgeries (27).

The core idea of ERAS is to reduce the stress of trauma. The postoperative recovery time is directly related to surgical stress. The previous study demonstrated that the patients undergoing full robot-assisted gastrectomy have an average 1-day reduction in a hospital stay as compared to patients undergoing robot-assisted gastrectomy with mini-laparotomy for anastomosis (28). In addition, robotic surgery can effectively reduce the trauma stress caused by surgery, thus reducing the negative response to stress and speeding up organ function recovery to achieve rapid recovery. However, there are certain demerits of robotic surgery, such as increased operation cost and extended operation time. Better short-term outcomes and technical advantages can offset these deficiencies.

In addition, the ultimate goal of ERAS is to reduce postoperative complications, reduce the length of the patient’s stay, and reduce the cost of hospitalization. Surgeons need to select the surgical techniques and instruments on an individual basis according to the patient’s situation, which is an important link in reducing surgical stress and postoperative complications.

Following are the limitations of our study: (1) due to the retrospective nature, we could not evaluate some important factors, such as cost-effectiveness and analysis of lymph nodes in different regions, (2) PSM could not offset all biases, and (3) the follow-up time of the patients is not long enough for us to collect clinical data. We could not compare the long-term oncology results between the two groups. Large-scale and multicenter randomized controlled trials (RCTs) should be conducted to confirm the reliability of the results.

In conclusion, RADG provides a short-term incidence of postoperative complications similar to that of LADG. RADG also showed less blood loss, an earlier anal exhaust, and a shorter postoperative hospitalization time. With a series of new implementation strategies, a robotic technique combined with ERAS protocol will be a better approach for patients with gastric cancer.

## Data Availability

The original contributions presented in the study are included in the article/Supplementary Material, further inquiries can be directed to the corresponding author.
